# Surgical Management of Small Bowel Crohn's Disease

**DOI:** 10.3389/fsurg.2022.759668

**Published:** 2022-04-15

**Authors:** Pramodh Chandrasinghe

**Affiliations:** Department of Surgery, Faculty of Medicine, University of Kelaniya, Ragama, Sri Lanka

**Keywords:** small bowel Crohn's, inflammatory strictures, fibrotic strictures, penetrating disease, stricturoplasty

## Abstract

Crohn's disease in the small bowel could present itself as an inflammatory stricture, a fibrotic stricture as penetrating disease or a combination of both. It is pertinent to differentiate the disease process as well as its extent to effectively manage the disease. Currently, a combination of medical and surgical therapies forms part of the treatment plan while the debate of which therapy is better continues. In managing the strictures, identification of the disease process through imaging plays a pivotal role as inflammatory strictures respond to anti-tumor necrosis factor (TNF) and biological agents, while fibrotic strictures require endoscopic or surgical intervention. Recent evidence suggests a larger role for surgical excision, particularly in ileocolic disease, while achieving a balance between disease clearance and bowel preservation. Several adaptations to the surgical technique, such as wide mesenteric excision, side to side or Kono-S anastomosis, and long-term metronidazole therapy, are being undertaken even though their absolute benefit is yet to be determined. Penetrating disease requires a broader multidisciplinary approach with a particular focus on nutrition, skincare, and intestinal failure management. The current guidance directs toward early surgical intervention for penetrating disease when feasible. Accurate preoperative imaging, medical management of active diseases, and surgical decision-making based on experience and evidence play a key role in success.

## Introduction

Some degree of involvement of the small bowel is seen in 70–80% of patients with Crohn's disease (CD), with approximately two-thirds having ileocolic involvement and another 10–30% having isolated small bowel involvement (1). Crohn's disease is of an unknown etiology, causing inflammation of the gastrointestinal tract from mouth to anus and the perineum. The prevalence of CD is high in Western Europe, Northern Europe, the USA, and Australia (2). Its etiopathogenesis is thought to be a combination of environmental exposure and genetic predisposition. The first encounter is understood to be the disruption of the epithelial barrier causing exposure of the luminal antigens to submucosal immune cells triggering an uncontrolled immune response in susceptible individuals (3, 4). The inflammation in CD is transmural, causing cryptitis, crypt abscesses, and epithelioid non-caseating granulomas.

There are mainly two types of small bowel CD: the stricturing type and the penetrating type. While the stricturing type gives rise to obstructive symptoms, the penetrating type causes fistulation or intra-abdominal sepsis. Surgical management of small bowel CD is an area of controversy and ambiguity, hence it is not commonly addressed. Although the treatment has to be personalized due to the varying nature of the disease, there are few important principles that govern the management of small bowel CD.

## STRICTURING Small Bowel CD

### Fibrotic vs. Inflammatory Strictures

There are two types of strictures in CD, and their identification is crucial for disease management. The excessive repair response to inflammation through the laying down of extracellular matrix (ECM) is postulated to cause luminal narrowing. The interplay between the inflammatory cells, the extracellular matrix, and the microbiota has been implicated in stricture formation, and the mechanisms overlap between both types of strictures (5, 6). In contrast to inflammatory strictures, the formation of fibrotic strictures is predominated by the accumulation of collagen-rich ECM that is principally produced by myofibroblasts under the influence of cytokines (7–9). While inflammatory strictures benefit from medical therapy, fibrotic strictures require endoscopic or surgical intervention.

### Imaging Modality

In imaging, a stricture is defined based on luminal narrowing (<50%), wall thickening (>25% or >3 mm), and pre-stricture dilation (>20% or >3 cm) compared to well distended adjacent normal bowel (9). MRI enterography (MRE) has become the investigation of choice due to its less invasive nature and the lack of radiation exposure (9). Contrast-enhanced CT (CECT) scans have the disadvantage of exposing patients to high doses of radiation, while capsule endoscopy carries the risk of being retained at a tight stricture (10). MRI has proven to be capable of accurately assessing whether the stricture is inflammatory or fibrotic in nature (11, 12). Wagner et al. (6) demonstrated a high level of correlation between MRI imaging and the histopathological composition of ileal strictures. However, in patients with acute intestinal obstruction, CECT would be the investigation of choice as MRE requires the ingestion of large amounts of oral contrast (9). Transabdominal ultrasound scans are equally effective in expert hands to visualize and monitor the progression of terminal ileal strictures due to their consistent anatomical location (13, 14). It has been shown to have a similar sensitivity to other imaging modalities with less exposure to radiation (15, 16). However, given that the quality of information is dependent on the operator, wider use of transabdominal ultrasound scans is limited (17).

## Endoscopic vs. Surgical Management

The goals of the therapy for strictures in small bowel CD are clinical symptom alleviation and radiological or endoscopic improvement. Strictures with a fibrostenotic component predicted by imaging have a lower threshold to be referred for surgical or endoscopic treatment, as none of the available medical therapies are effective against fibrosis (18, 19).

Both endoscopic balloon dilatation (EBD) and surgery are acceptable modalities of treatment for CD strictures (18–21). It is important to map out the entire small bowel prior to deciding on the type of intervention. However, it is not uncommon to find previously undetected pathological segments during the procedure. Balloon expansion should aim for a size up to 18–20 mm, even with several attempts, in order to gain a maximum surgery-free survival (18). Visualization of cracking through the balloon is recommended, and attempts should be made to travel through the stricture with the scope following dilatation. There is no consensus on the holding time for expanded balloon (18). The efficacy of EBD has been shown to reduce with strictures longer than 4 cm in length at a rate of 8% for each additional cm (19, 22). There is much debate on the timing of surgical intervention in Crohn's disease strictures (23). Early surgical intervention, especially for ileocolic disease, has been proposed following emerging evidence from the LIR!C trial (24–27). This randomized trial demonstrated that early laparoscopic ileocolic resection in patients with poor response to conventional Crohn's medication gave an equal quality of life with less medical therapy at a significantly lower cost. Additionally, some expert guidance recommend surgery without biologic trials for fibrotic strictures (28).

## Stricturoplasty VS. Small Bowel Resection

Conventionally, small bowel resection was considered the last resort due to the risk of short bowel syndrome, high rates of anastomotic leakage, and intra-abdominal collections, especially in patients who have been on a long-term steroid therapy. Of late, few investigators have challenged this conventional understanding given the comparable outcome and cost. Some even question whether the role of medical therapy is to delay the eventuality of surgery (25, 27). Several recent studies suggest surgical excision to provide a better quality of life and a significant reduction in cost compared to anti-TNF therapy in isolated ileocolic disease (25, 29).

Stricturoplasty for CD has the advantage of preserving small bowel length, especially in those with multiple sites of obstructions. Traditionally, stricturoplasty was recommended for strictures <10 cm in length (30). However, innovative approaches, such as Finney and Michelassi techniques, allow for much longer segments to be managed. The simplest type is the Heiniken-Mickulicz technique, which includes a longitudinal enterotomy made across the stricture segment and closed transversely (30). The Finney technique is done by folding the diseased bowel on itself and creating a large opening between the two limbs of the loop to overcome the obstruction. This method leaves a longer suture line through the diseased bowel segment, increasing the chances of complications. The Michelassi procedure, which is a complex procedure for long-segment strictures, involves dividing the stricture in the middle and performing a side-to-side anastomosis in an isoperistaltic orientation (31). Stricturoplasty has a similar long-term outcome compared to resection and is specifically recommended for multiple strictures, previous long-segment resections, early recurrences, short bowel syndrome, and those with malnutrition (32). In addition to conventional procedures, several non-conventional stricturoplasties have also been done across the ileocecal valve with comparable outcomes (33).

Commonly, patients are managed with a combination of resection and stricturoplasty for the remaining segments. The approach depends on the individual case after considering multiple factors such as patient's age, fitness, chances of recurrence, availability of medical therapy, and severity of the disease. Quite interestingly, the site-specific recurrence rates have shown to be 2–5% in 10 years along with the normalized bowel wall during re-surgery (34). European guidance also recommends stricturoplasty as the first option for surgical intervention in CD when technically feasible.

## Wide Mesenteric vs. Close Dissection

Coffey et al. (35) reported a 2.9% recurrence rate in wide mesenteric resection compared to a 40% recurrence rate in conventional close dissection (36). They hypothesized that the primary pathology in small bowel CD initiates from the mesentery. Therefore, removal of the mesentery was proposed to prevent disease recurrence (37). However, others who hypothesize that the confounding factor is resection margin involvement rather than the mesenteric excision have challenged their results (38). A randomized controlled trial to test the efficacy of wide mesenteric excision is currently underway (39). Currently, the use of frozen sections to define the resection margins is not followed, and it is recommended that the macroscopically disease-free region is identified. Those who oppose the novel concept of wide mesenteric resection argue that, with stricturoplasty having similar long-term results to resectional surgery disproves the importance of the mesentery in disease recurrence (40). Regardless of the pre-operative imaging, most surgeons prefer to check for unforeseen strictures both upstream and downstream by passing a Foley catheter with its bulb inflated up to 2 cm in diameter. It is also pertinent to note the remaining length of the small bowel after the resection. The laparoscopic approach has proven to be safe and feasible with minimal access trauma and is also recommended by the European guidelines whenever possible. The single incision laparoscopic surgical (SILS) technique has also proven to be effective since it allows the easy exteriorization of the diseased segment through the port to perform complex procedures. Reduced adhesion formation allowing easier repeated surgeries is a perceived benefit of the minimal access surgery in CD (41).

## Biologics and Steroids in the Perioperative Period

There is no consensus on the preoperative management of steroids and anti-TNF therapy. A steroid dose of 20 mg and more for a period exceeding 6 weeks increases post-operative complications and should be weaned if possible without increasing the disease burden (42, 43). Evidence on the requirement of a stress dose of steroids preoperatively is also poor, and there is no consensus on such modification vs. continuation of the normal dose. There is conflicting evidence on the increased postoperative complication rates when providing anti-TNF therapy in the perioperative period although few recent meta analyses demonstrate a higher rate of complication (42, 44–47). However, the optimal drug-free interval prior to surgery in order to minimize complications is not known. Optimization of nutrition, correction of hemoglobin levels, and resolution of intra-abdominal septic foci prior to surgical intervention are also of prime importance to reduce postoperative anastomotic complications (48–50).

## Preventing Recurrences

All attempts are taken to prevent an anastomotic recurrence following bowel resection. A wide side-to-side (functional end to end) anastomosis is recommended (42, 51). Neither stapled nor hand-sewn techniques have shown an advantage over the other. Kono-S is another innovative anastomotic technique recently adopted for CD following the hypothesis that disease causation is linked to the mesentery (52, 53). This technique aims to keep the suture line away from the mesentery. Although several previous studies have shown lower rates of recurrent strictures with this technique, there is not enough evidence to draw clear conclusions. The use of low doses of oral metranidazole for 3 months following surgery has been shown to reduce recurrent strictures (54). It is recommended that patients start anti-TNF therapy within 2 weeks of surgery to gain maximum re-surgery-free survival (42). The traditional viewpoint has been to delay the start of biological agents in the postoperative period due to fears of delayed wound failure and infection (55, 56). However, there is convincing new evidence for the early initiation of biological agents and their positive effects on preventing recurrences without additional risk of infection (57, 58). Lightner et al. (57) recently reported comparable short-term outcomes in patients who had biologics restarted within 90 days of surgery. There is evidence to suggest that most recently available biologics can be used safely during the perioperative period (59–61).

## Penetrating Small Bowel CD

The transmural inflammation in CD results in fistulation. Small bowel fistulae, a difficult entity to manage, can occur at any site between the duodenum and the terminal ileum. The pathophysiology of the disease is largely unknown although TGFβ appears to play a role in stimulating epithelial–mesenchymal transition (62). Fistulation could be enterocutaneous (ECF), entero-enteric (EEF), or entero-vesicular (EVF).

ECF accounts for around 5% of the fistulizing CD (63) and can be seen arising either *de novo* or postoperatively. The fistula output will depend on the site with more proximal fistulae giving rise to troublesome high outputs. Post-procedural ECF presents itself within the early postoperative period as an abscess under the scar or a feculent discharge through the scar, the drain site, or the port site following laparoscopic surgery. *De-novo* fistulae also manifest initially as an abscess, which continues to discharge the following drainage. They may also track from an intervening abscess cavity between the bowel and the skin commonly in the iliac fossa or the pelvis (64).

**Figure 1:**
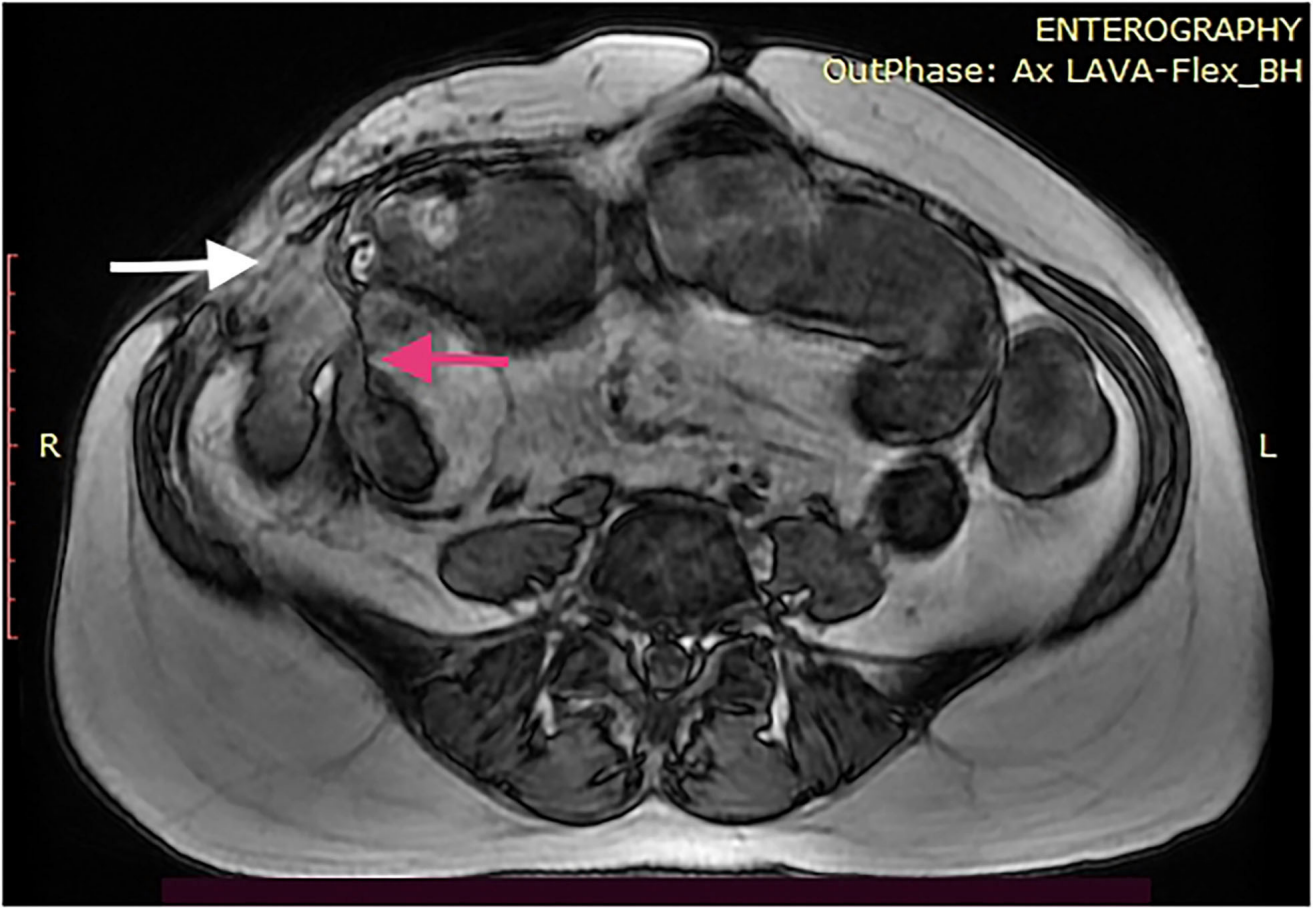
MRI enterography of a 46-year-old woman with CD with an enterocutaneous fistula (ECF) at the right iliac fossa (white arrow). She has undergone two ileocolic resections previously and a stricturoplasty of the end to end anastomosis within a 7-year period. The image demonstrates proximal bowel dilatation and a possible distal stricture (red arrow).

**Figure 2:**
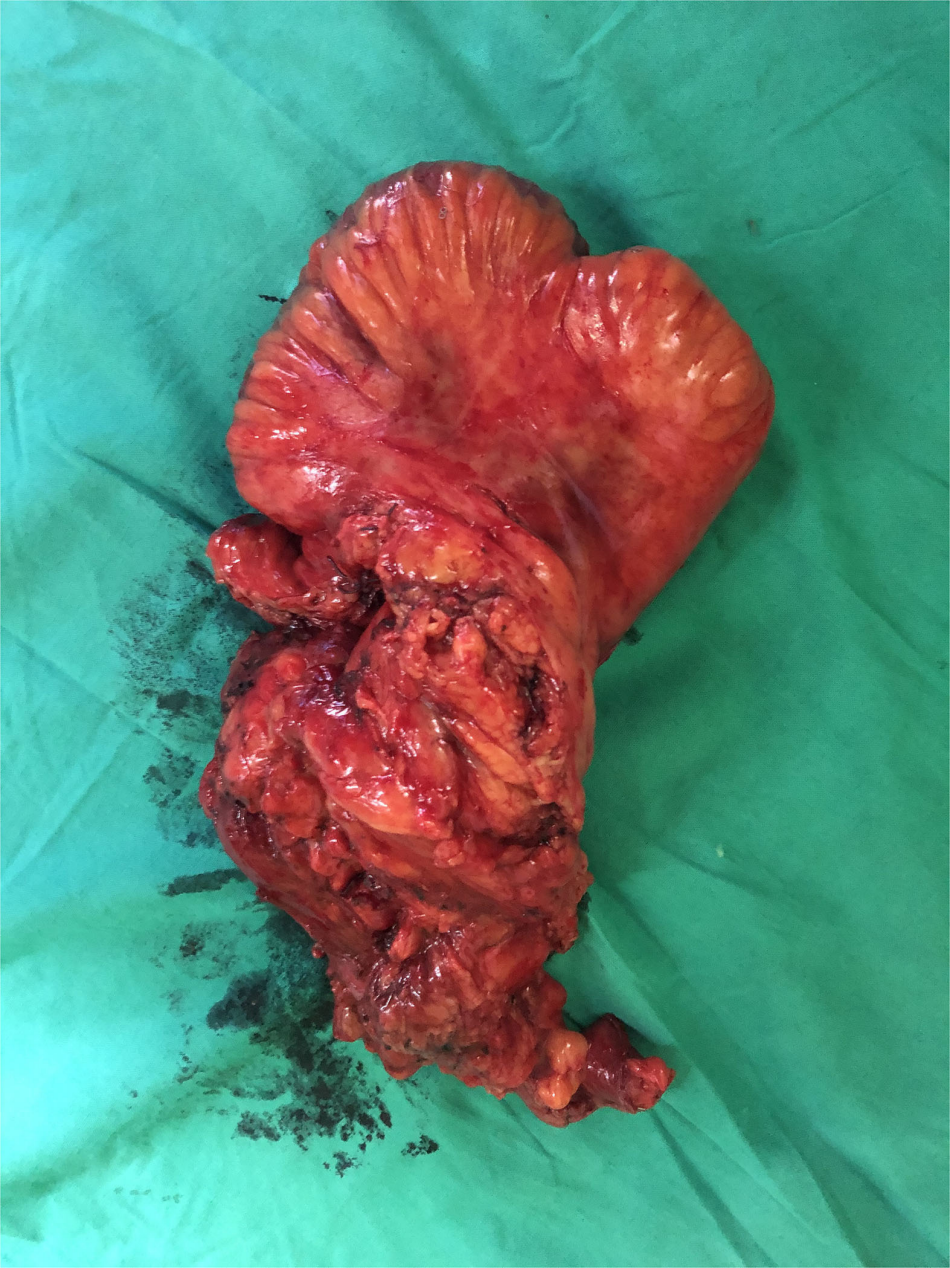
Resection of the ileal segment involved (shown in Figure 1) in the ECF with a wide mesenteric margin. A side-to-side (functional end to end) stapled anastomosis was performed to achieve bowel continuity. Significant inflammatory changes were observed in the mesentery. The patient was prescribed low-dose metronidazole for 3 months. The patient is asymptomatic to date.

CT and MRI are the preferred imaging modalities to define the tracks anatomically (Figure 1). Any septic foci need to be drained urgently either surgically or radiologically and the fistula tract needs to be addressed after a thorough assessment. The entire small bowel needs to be assessed for strictures distally along with the extent of disease involvement. Maintaining nutrition, skincare, and the control of sepsis play a major role in managing ECF, indicating the importance of a multidisciplinary setting for best outcomes. A low output fistula may spontaneously close with initial bowel rest and nutritional support. *De-novo* ECF might respond to biological therapy and elemental feeds; however, operative intervention is indicated early to prevent complications (42). The data on the effect of biologics on fistulating Crohn's disease needs to be interpreted with caution. Most studies have a heterogenic patient population, which includes patients with anal fistula (65). Amiot et al. (66) retrospectively comparing surgery vs. biologics in ECF reported that 54% required surgical intervention. The authors concluded that anti-TNF therapy might have a place in managing a simple fistula without distal stenosis. Medical management has no place in post-procedure ECF if there is no active disease. Surgery for most ECF will include removing the fistula, resecting the involved bowel segment, and ensuring the absence of strictures distally (Figure 2). At times, a staged procedure may be performed by exteriorizing the bowel ends and controlling the sepsis prior to restoring continuity.

EEF is not commonly discussed. Its presentation may vary depending on the site of the fistulae. Entero-colic fistulae (fistula between small bowel and colon) might present with diarrhea while an EEF between close segments of the small bowel may be asymptomatic. A resulting blind loop may give rise to bacterial overgrowth. Patients with EEF require personalized treatment in a multidisciplinary setting (64). Most EEF can be managed by disconnecting the fistula and primary repair. A short segment of small bowel involvement can be managed through resection of the segment and restoration of continuity. Laparoscopic surgery may be feasible in this setting although a high rate of conversion is to be expected (45).

In CD, perforations in the small bowel commonly occur at the terminal ileum due to penetrating disease (67, 68). Patients complain of pain in the right iliac fossa and experience tenderness, usually associated with fever. Most patients with perforated small bowel CD will not require operative management. The perforation is mostly walled off as an abscess or a mass and is best managed conservatively. Interventions in the acute stage might increase the risk of developing post-procedure ECF. A CECT scan with oral contrast is highly sensitive to diagnose the perforation, which will demonstrate extravasation of contrast along with surrounding free gas is pathognomonic. If a significant fluid collection is present, radiological guided drainage and insertion of a tube drain are preferred. Intravenous antibiotic therapy will be effective in the acute stage followed by disease-modifying medication to control active diseases prior to surgery. Especially, isolated ileocolic disease responds well to surgery.

Entero-vesicular accounts for a small portion of penetrating disease in CD, with an incidence of ~2% of all fistulae (69). EVF could be simple with a single bowel loop penetrating the bladder or be complex with a bowel mass or an intervening abscess penetrating the bladder. Early surgery is recommended in EVF cases as complete closure is unlikely with medical therapy and could become complicated with sepsis. Other abnormal communications such as entero-vaginal or entero-urethral fistula can also occur requiring specialized disease management (64).

Chron's disease in the small bowel requires multiple surgical interventions, potentially resulting in intestinal failure (IF). An audit conducted in the UK demonstrated that ~25% of patients with IF in CD were due to repeated bowel resections (70). Therefore, it is important that CD of the small bowel is managed in a multi-disciplinary setting to facilitate informed decisions on preserving the bowel length as much as possible and manage IF in the event of such a complication. Specialized centers may offer bowel-lengthening procedures such as transverse enteroplasties.

## Conclusion

Crohn's disease in the small bowel requires comprehensive care in a multidisciplinary setting. Classifying the disease based on its pathology into inflammatory strictures or fibriotic strictures plays a major role in disease management. While inflammatory CD can be managed with medical therapy, surgery has become the mainstay for strictures in CD with good outcomes. Bowel resection with or without wide mesenteric excision and multiple stricturoplasty or a combination of both is undertaken and offers similar outcomes. A combination of medical and surgical therapy is required in most cases, while the debate on their effectiveness as stand-alone treatment plans continues. Penetrating disease in small bowel CD is challenging, and surgery is recommended early to prevent complications. They require meticulous preoperative planning and optimization in a specialized care setting. The consequences of small bowel CD, such as intestinal failure, require highly specialized disease management with enteral nutrition and corrective procedures.

## Author Contributions

PC was involved in the conceptual planning, writing manuscript and critically appraising the final draft.

## Conflict of Interest

The author declares that the research was conducted in the absence of any commercial or financial relationships that could be construed as a potential conflict of interest.

## Publisher's Note

All claims expressed in this article are solely those of the authors and do not necessarily represent those of their affiliated organizations, or those of the publisher, the editors and the reviewers. Any product that may be evaluated in this article, or claim that may be made by its manufacturer, is not guaranteed or endorsed by the publisher.

## References

[1] Baumgart DC Sandborn WJ. Crohn's disease. Lancet. (2012) 380:1590–605. 10.1016/S0140-6736(12)60026-9 22914295

[2] Shivashankar R Tremaine WJ Harmsen WS Loftus EV. Incidence and prevalence of Crohn's disease and ulcerative colitis in Olmsted County, minnesota from 1970 through 2010. Clin Gastroenterol Hepatol. (2017) 15:857–63. 10.1016/j.cgh.2016.10.039 27856364 PMC5429988

[3] Thoo L Noti M Krebs P. Keep calm: the intestinal barrier at the interface of peace and war. Cell Death Dis. (2019) 10:849. 10.1038/s41419-019-2086-z 31699962 PMC6838056

[4] Lechuga S Ivanov AI. Disruption of the epithelial barrier during intestinal inflammation: Quest for new molecules and mechanisms. Biochim Biophys Acta Mol Cell Res. (2017) 1864:1183–94. 10.1016/j.bbamcr.2017.03.007 28322932 PMC5507344

[5] Rieder F Fiocchi C Rogler G. Mechanisms, management, and treatment of fibrosis in patients with inflammatory bowel diseases. Gastroenterology. (2017) 152:340–50 e6. 10.1053/j.gastro.2016.09.047 27720839 PMC5209279

[6] Wagner M Ko HM Chatterji M Besa C Torres J Zhang X. Magnetic resonance imaging predicts histopathological composition of ileal Crohn's disease. J Crohns Colitis. (2018) 12:718–29. 10.1093/ecco-jcc/jjx186 29300851 PMC7189968

[7] Stallmach A Schuppan D Riese HH Matthes H Riecken EO. Increased collagen type III synthesis by fibroblasts isolated from strictures of patients with Crohn's disease. Gastroenterology. (1992) 102:1920–9. 10.1016/0016-5085(92)90314-O 1587410

[8] Scheibe K Kersten C Schmied A Vieth M Primbs T Carle B. Inhibiting interleukin 36 receptor signaling reduces fibrosis in mice with chronic intestinal inflammation. Gastroenterology. (2019) 156:1082–97 e11. 10.1053/j.gastro.2018.11.029 30452921

[9] Bettenworth D Bokemeyer A Baker M Mao R Parker CE Nguyen T. Assessment of Crohn's disease-associated small bowel strictures and fibrosis on cross-sectional imaging: a systematic review. Gut. (2019) 68:1115–26. 10.1136/gutjnl-2018-318081 30944110 PMC6580870

[10] Estay C Simian D Lubascher J Figueroa C O'Brien A Quera R. Ionizing radiation exposure in patients with inflammatory bowel disease: are we overexposing our patients? J Dig Dis. (2015) 16:83–9. 10.1111/1751-2980.12213 25420751

[11] Mentzel HJ Reinsch S Kurzai M Stenzel M. Magnetic resonance imaging in children and adolescents with chronic inflammatory bowel disease. World J Gastroenterol. (2014) 20:1180–91. 10.3748/wjg.v20.i5.1180 24574794 PMC3921502

[12] Masselli G Colaiacomo MC Marcelli G Bertini L Casciani E Laghi F. MRI of the small-bowel: how to differentiate primary neoplasms and mimickers. Br J Radiol. (2012) 85:824–37. 10.1259/bjr/14517468 22422388 PMC3474119

[13] Panes J Bouhnik Y Reinisch W Stoker J Taylor SA Baumgart DC. Imaging techniques for assessment of inflammatory bowel disease: joint ECCO and ESGAR evidence-based consensus guidelines. J Crohns Colitis. (2013) 7:556–85. 10.1016/j.crohns.2013.02.020 23583097

[14] Strobel D Goertz RS Bernatik T. Diagnostics in inflammatory bowel disease: ultrasound. World J Gastroenterol. (2011) 17:3192–7. 21912467 10.3748/wjg.v17.i27.3192 PMC3158394

[15] Panes J Bouzas R Chaparro M Garcia-Sanchez V Gisbert JP Martinez de. Guerenu B. Systematic review: the use of ultrasonography, computed tomography and magnetic resonance imaging for the diagnosis, assessment of activity and abdominal complications of Crohn's disease. Aliment Pharmacol Ther. (2011) 34:125–45. 10.1111/j.1365-2036.2011.04710.x 21615440

[16] Castiglione F Mainenti PP De Palma GD Testa A Bucci L Pesce G. Noninvasive diagnosis of small bowel Crohn's disease: direct comparison of bowel sonography and magnetic resonance enterography. Inflamm Bowel Dis. (2013) 19:991–8. 10.1097/MIB.0b013e3182802b87 23429465

[17] Asthana AK Friedman AB Maconi G Maaser C Kucharzik T Watanabe M. Failure of gastroenterologists to apply intestinal ultrasound in inflammatory bowel disease in the Asia-Pacific: a need for action. J Gastroenterol Hepatol. (2015) 30:446–52. 10.1111/jgh.12871 25529767

[18] Shen B Kochhar G Navaneethan U Farraye FA Schwartz DA Iacucci M. Practical guidelines on endoscopic treatment for Crohn's disease strictures: a consensus statement from the Global Interventional Inflammatory Bowel Disease Group. Lancet Gastroenterol Hepatol. (2020) 5:393–405. 10.1016/S2468-1253(19)30366-8 31954438

[19] Lan N Stocchi L Ashburn JH Hull TL Steele SR Delaney CP. Outcomes of endoscopic balloon dilation vs surgical resection for primary ileocolic strictures in patients with Crohn's disease. Clin Gastroenterol Hepatol. (2018) 16:1260–7. 10.1016/j.cgh.2018.02.035 29505909

[20] Steinhardt HJ Loeschke K Kasper H Holtermuller KH Schafer H. European Cooperative Crohn's Disease Study (ECCDS): clinical features and natural history. Digestion. (1985) 31:97–108. 10.1159/000199186 2860044

[21] Hirai F Andoh A Ueno F Watanabe K Ohmiya N Nakase H. Efficacy of endoscopic balloon dilation for small bowel strictures in patients with Crohn's disease: a nationwide, multi-centre, open-label, prospective cohort study. J Crohns Colitis. (2018) 12:394–401. 10.1093/ecco-jcc/jjx159 29194463

[22] Bettenworth D Gustavsson A Atreja A Lopez R Tysk C van Assche G. A Pooled analysis of efficacy, safety, and long-term outcome of endoscopic balloon dilation therapy for patients with stricturing Crohn's disease. Inflamm Bowel Dis. (2017) 23:133–42. 10.1097/MIB.0000000000000988 28002130

[23] An V Cohen L Lawrence M Thomas M Andrews J Moore J. Early surgery in Crohn's disease a benefit in selected cases. World J Gastrointest Surg. (2016) 8:492–500. 10.4240/wjgs.v8.i7.492 27462391 PMC4942749

[24] Ponsioen CY de Groof EJ Eshuis EJ Gardenbroek TJ Bossuyt PMM Hart A. Laparoscopic ileocaecal resection versus infliximab for terminal ileitis in Crohn's disease: a randomised controlled, open-label, multicentre trial. Lancet Gastroenterol Hepatol. (2017) 2:785–92. 10.1016/S2468-1253(17)30248-0 28838644

[25] de Groof EJ Stevens TW Eshuis EJ Gardenbroek TJ Bosmans JE van Dongen JM. Cost-effectiveness of laparoscopic ileocaecal resection versus infliximab treatment of terminal ileitis in Crohn's disease: the LIR!C Trial. Gut. (2019) 68:1774–80. 10.1136/gutjnl-2018-317539 31233395

[26] de Groof EJ Gardenbroek TJ Buskens CJ Tanis PJ Ponsioen CY D'Haens G. The association between intensified medical treatment, time to surgery and ileocolic specimen length in Crohn's disease. Colorectal Dis. (2017) 19:551–8. 10.1111/codi.13567 27883259

[27] Stevens TW Haasnoot ML D'Haens GR Buskens CJ de Groof EJ Eshuis EJ. Laparoscopic ileocaecal resection versus infliximab for terminal ileitis in Crohn's disease: retrospective long-term follow-up of the LIR!C trial. Lancet Gastroenterol Hepatol. (2020) 5:900–7. 10.1016/S2468-1253(20)30117-5 32619413

[28] Meima-van Praag EM Buskens CJ Hompes R Bemelman WA. Surgical management of Crohn's disease: a state of the art review. Int J Colorectal Dis. (2021) 36:1133-45. 10.1007/s00384-021-03857-2 33528750 PMC8119249

[29] Broide E Eindor-Abarbanel A Naftali T Shirin H Shalem T Richter V. Early Surgery versus biologic therapy in limited nonstricturing ileocecal Crohn's disease-a decision-making analysis. Inflamm Bowel Dis. (2020) 26:1648–57. 10.1093/ibd/izz282 31909420

[30] Ambe R Campbell L Cagir B.A comprehensive review of strictureplasty techniques in Crohn's disease: types, indications, comparisons, and safety. J Gastrointest Surg. (2012) 16:209–17. 10.1007/s11605-011-1651-2 21909847

[31] Michelassi F. Side-to-side isoperistaltic strictureplasty for multiple Crohn's strictures. Dis Colon Rectum. (1996) 39:345–9. 10.1007/BF02049480 8603560

[32] Sampietro GM Corsi F Maconi G Ardizzone S Frontali A Corona A. Prospective study of long-term results and prognostic factors after conservative surgery for small bowel Crohn's disease. Clin Gastroenterol Hepatol. (2009) 7:183–91. 10.1016/j.cgh.2008.10.008 19118641

[33] Sampietro GM Cristaldi M Maconi G Parente F Sartani A Ardizzone S. A prospective, longitudinal study of nonconventional strictureplasty in Crohn's disease. J Am Coll Surg. (2004) 199:8–20. 10.1016/j.jamcollsurg.2004.01.039 15217622

[34] Brown SR Fearnhead NS Faiz OD Abercrombie JF Acheson AG Arnott RG. The Association of Coloproctology of Great Britain and Ireland consensus guidelines in surgery for inflammatory bowel disease. Colorectal Dis. (2018) 20 Suppl 8:3–117. 10.1111/codi.14448 30508274

[35] Coffey CJ Kiernan MG Sahebally SM Jarrar A Burke JP Kiely PA. Inclusion of the mesentery in ileocolic resection for Crohn's disease is associated with reduced surgical recurrence. J Crohns Colitis. (2018) 12:1139–50. 10.1093/ecco-jcc/jjx187 29309546 PMC6225977

[36] van der Does de. Willebois EML Buskens CJ Bemelman WA. Technical standardization of laparoscopic extended mesenterectomy in ileocolic resection in Crohn's disease - a video vignette. Colorectal Dis. (2021) 23:1015–6. 10.1111/codi.15546 33503309

[37] Coffey JC O'Leary DP. The mesentery: structure, function, and role in disease. Lancet Gastroenterol Hepatol. (2016) 1:238–47. 10.1016/S2468-1253(16)30026-7 28404096

[38] Caprino P Sacchett F Sofo L. A warning about the role of extended mesenteric excision in crohn's disease recurrence. J Crohn's Colitis. (2019) 13:1583. 10.1093/ecco-jcc/jjz097 31076765

[39] Li Y Mohan H Lan N Wu X Zhou W Gong J. Mesenteric excision surgery or conservative limited resection in Crohn's disease: study protocol for an international, multicenter, randomized controlled trial. Trials. (2020) 21:210. 10.1186/s13063-020-4105-x 32085793 PMC7035646

[40] Butt WT Ryan EJ Boland MR McCarthy EM Omorogbe J Hazel K. Strictureplasty versus bowel resection for the surgical management of fibrostenotic Crohn's disease: a systematic review and meta-analysis. Int J Colorectal Dis. (2020) 35:705–17. 10.1007/s00384-020-03507-z 32048011

[41] Leo CA Samaranayake SF Chandrasinghe PC Shaikh IA Hodgkinson JD Warusavitarne JH. Single Port Laparoscopic Surgery for Complex Crohn's Disease Is Safe with a Lower Conversion Rate. J Laparoendosc Adv Surg Tech A. (2017) 27:1095–100. 10.1089/lap.2016.0567 28475480

[42] Bemelman WA Warusavitarne J Sampietro GM Serclova Z Zmora O Luglio G. ECCO-ESCP consensus on surgery for Crohn's Disease. J Crohns Colitis. (2018) 12:1–16. 10.1093/ecco-jcc/jjx061 28498901

[43] Gionchetti P Dignass A Danese S Magro Dias FJ Rogler G Lakatos PL. 3rd European Evidence-based Consensus on the Diagnosis and Management of Crohn's Disease 2016: Part 2: Surgical Management and Special Situations. J Crohns Colitis. (2017) 11:135–49. 10.1093/ecco-jcc/jjw169 27660342

[44] Quaresma AB Yamamoto T Kotze PG. Biologics and surgical outcomes in Crohn's disease: is there a direct relationship? Therap Adv Gastroenterol. (2020) 13:1756284820931738. 10.1177/1756284820931738 32728389 PMC7366403

[45] Kristo I Stift A Argeny S Mittlbock M Riss S. Minimal-invasive approach for penetrating Crohn's disease is not associated with increased complications. Surg Endosc. (2016) 30:5239–44. 10.1007/s00464-016-4871-4 27334961 PMC5112282

[46] Kotze PG Ghosh S Bemelman WA Panaccione R. Preoperative use of anti-tumor necrosis factor therapy in Crohn's disease: promises and pitfalls. Intest Res. (2017) 15:160–5. 10.5217/ir.2017.15.2.160 28522944 PMC5430006

[47] Kulaylat AS Kulaylat AN Schaefer EW Tinsley A Williams E Koltun W. Association of preoperative anti-tumor necrosis factor therapy with adverse postoperative outcomes in patients undergoing abdominal surgery for ulcerative colitis. JAMA Surg. (2017) 152:e171538. 10.1001/jamasurg.2017.1538 28614561 PMC5831468

[48] Zangenberg MS Horesh N Kopylov U El-Hussuna A. Preoperative optimization of patients with inflammatory bowel disease undergoing gastrointestinal surgery: a systematic review. Int J Colorectal Dis. (2017) 32:1663–76. 10.1007/s00384-017-2915-4 29051981

[49] Sharma A Chinn BT. Preoperative optimization of crohn disease. Clin Colon Rectal Surg. (2013) 26:75–9. 10.1055/s-0033-1348044 24436653 PMC3710023

[50] Efron JE Young-Fadok TM. Preoperative optimization of Crohn's disease. Clin Colon Rectal Surg. (2007) 20:303–8. 10.1055/s-2007-991029 20011426 PMC2780221

[51] Feng JS Li JY Yang Z Chen XY Mo JJ Li SH. Stapled side-to-side anastomosis might be benefit in intestinal resection for Crohn's disease: a systematic review and network meta-analysis. Medicine (Baltimore). (2018) 97:e0315. 10.1097/MD.0000000000010315 29642162 PMC5908623

[52] Horisberger K Birrer DL Rickenbacher A Turina M. Experiences with the Kono-S anastomosis in Crohn's disease of the terminal ileum-a cohort study. Langenbecks Arch Surg (. (2020). 10.1007/s00423-020-01998-6 33025079 PMC8208918

[53] Alshantti A Hind D Hancock L Brown SR. The role of Kono-S anastomosis and mesenteric resection in reducing recurrence after surgery for Crohn's disease: a systematic review. Colorectal Dis. (2021) 23:7–17. 10.1111/codi.15136 32418300

[54] Glick LR Sossenheimer PH Ollech JE Cohen RD Hyman NH Hurst RD. Low-dose metronidazole is associated with a decreased rate of endoscopic recurrence of Crohn's disease after ileal resection: a retrospective cohort study. J Crohns Colitis. (2019) 13:1158–62. 10.1093/ecco-jcc/jjz047 30809655 PMC6939874

[55] Cohen-Mekelburg S Gold S Schneider Y Dennis M Oromendia C Yeo H. Delays in initiating post-operative prophylactic biologic therapy are common among Crohn's disease patients. Dig Dis Sci. (2019) 64:196–203. 10.1007/s10620-018-5159-4 29876778

[56] Fields AC Melnitchouk N. Medical prophylaxis of post-surgical crohn's disease recurrence: towards timely anti-TNF therapy. Dig Dis Sci. (2019) 64:7–8. 10.1007/s10620-018-5236-8 30097893

[57] Lightner AL Grass F Alsughayer AM Harmsen WS Petersen M Loftus EV. Are biologics safe in the immediate postoperative period? A single-center evaluation of consecutive Crohn's surgical patients. Dis Colon Rectum. (2020) 63:934–43. 10.1097/DCR.0000000000001649 32149787

[58] Lightner AL. Perioperative management of biologic and immunosuppressive medications in patients with Crohn's disease. Dis Colon Rectum. (2018) 61:428–31. 10.1097/DCR.0000000000001072 29521822

[59] Shim HH Ma C Kotze PG Seow CH Al-Farhan H Al-Darmaki AK. Preoperative ustekinumab treatment is not associated with increased postoperative complications in Crohn's disease: a canadian multi-centre observational cohort study. J Can Assoc Gastroenterol. (2018) 1:115–23. 10.1093/jcag/gwy013 31294352 PMC6507292

[60] Wright EK Kamm MA De Cruz P Hamilton AL Selvaraj F Princen F. Anti-TNF Therapeutic Drug Monitoring in Postoperative Crohn's Disease. J Crohns Colitis. (2018) 12:653–61. 10.1093/ecco-jcc/jjy003 29385469

[61] Xu Y Yang L An P Zhou B Liu G. Meta-Analysis: The influence of preoperative infliximab use on postoperative complications of Crohn's disease. Inflamm Bowel Dis. (2019) 25:261–9. 10.1093/ibd/izy246 30052982

[62] Scharl M Rogler G. Pathophysiology of fistula formation in Crohn's disease. World J Gastrointest Pathophysiol. (2014) 5:205–12. 10.4291/wjgp.v5.i3.205 25133023 PMC4133520

[63] Schwartz DA Maltz BE. Treatment of fistulizing inflammatory bowel disease. Gastroenterol Clin North Am. (2009) 38:595–610. 10.1016/j.gtc.2009.07.009 19913204

[64] Lightner AL Ashburn JH Brar MS Carvello M Chandrasinghe P van Overstraeten AB. Fistulizing Crohn's disease. Curr Probl Surg. (2020) 57:100808. 10.1016/j.cpsurg.2020.100808 33187597

[65] Ma C Parker CE Nguyen TM Khanna R Feagan BG Jairath V. Identifying outcomes in clinical trials of fistulizing Crohn's disease for the development of a core outcome set. Clin Gastroenterol Hepatol. (2019) 17:1904–8. 10.1016/j.cgh.2018.10.004 30292887

[66] Amiot A Setakhr V Seksik P Allez M Treton X De Vos M. Long-term outcome of enterocutaneous fistula in patients with Crohn's disease treated with anti-TNF therapy: a cohort study from the GETAID. Am J Gastroenterol. (2014) 109:1443–9. 10.1038/ajg.2014.183 25091063

[67] Philip S Kamyab A Orfanou P. A free terminal ileal perforation from active crohn disease in pregnancy: a diagnostic challenge. Int Surg. (2015) 100:450–4. 10.9738/INTSURG-D-14-00070.1 25785326 PMC4370534

[68] Haddad A Ben Mahmoud A Chaker Y Zehani A Ksantini R Kacem MJ. Presentation with perforation of the terminal ileum and acute limb ischemia in Crohn's disease: a case report. Int J Surg Case Rep. (2021) 80:105626. 10.1016/j.ijscr.2021.02.012 33601328 PMC7898057

[69] Kaimakliotis P Simillis C Harbord M Kontovounisios C Rasheed S Tekkis PP. Systematic review assessing medical treatment for rectovaginal and enterovesical fistulae in Crohn's disease. J Clin Gastroenterol. (2016) 50:714–21. 10.1097/MCG.0000000000000607 27466166

[70] Agwunobi AO Carlson GL Anderson ID Irving MH Scott NA. Mechanisms of intestinal failure in Crohn's disease. Dis Colon Rectum. (2001) 44:1834–7. 10.1007/BF02234463 11742170

